# Risk Factors for Surgical Site Infections and the Effects of Betadine Irrigation and Intrawound Vancomycin Powder on Infection Rates in Spine Tumor Surgery

**DOI:** 10.7759/cureus.64591

**Published:** 2024-07-15

**Authors:** Addisu Mesfin, Mina Botros, Lancelot Benn, Andrea Kulp

**Affiliations:** 1 Department of Orthopedics Surgery, MedStar Washington Hospital Center, Washington, USA; 2 Department of Orthopedics & Physical Performance, University of Rochester Medical Center, Rochester, USA

**Keywords:** intrawound vancomycin powder, betadine, spine tumor, cancer, surgical site infection, infection, metastatic spine tumor

## Abstract

Background

Surgical site infection (SSI) following spine tumor surgery results in delays in radiation therapy and the initiation of systemic treatment. The study aims to assess risk factors for SSI in malignancy-related spinal infections and rates of infection observed in a single center with the use of betadine irrigation (BI) and intrawound vancomycin powder (IVP).

Methods

Spine tumor patients managed from 11/2012 to 11/2023 were identified using a surgical database (JotLogs, Efficient Surgical Apps, Portland, Maine). Inclusion criteria were patients receiving BI and IVP and alive at 30 days post-op. Exclusion criteria were patients not receiving a combination of BI and IVP due to allergies and mortality within 30 days of surgery. Patient demographics, histology, history of pre-operative and post-operative radiation treatment history, tumor location, procedure type, number of procedures per patient, SSI, wound culture results, and mortality were collected.

Results

One hundred two patients undergoing 130 procedures had an SSI rate of 3.85% (5/130). There were 18.6% primary and 81.4% metastatic tumors. Demographics were average age 59.5 years old (range 7-92), 60.8% male, 39.2% female, White 88.2%, Black 9.8%, and others 2%. Pre-operative radiation therapy was significantly associated with the risk of SSI (p=0.005). Percutaneous instrumentation did not lead to a significant difference in infection rates (p=0.139). There was no significant difference in infection rates between primary and metastatic tumors (p=0.58). Multivariable regression analysis revealed pre-operative radiation (OR: 18.1; 95%CI: 1.9-172.7; p=0.009) as the statistically significant independent risk factor.

Conclusions

Pre-operative radiation therapy remains a risk factor for SSI. However, percutaneous instrumentation did not lead to SSI, and there was no significant difference in infection rates between primary and metastatic tumors. SSI rate was 3.85% in patients who had a combination of BI and IVP in spine tumor surgery.

## Introduction

Spine tumor surgery has historically been associated with a high rate of surgical site infections (SSIs) ranging from 5% to 35% [1,2]. Risk factors for SSI in spinal tumor patients include pre-operative radiation therapy, malnutrition, steroid use, and history of smoking [1,2]. Consequences of SSI following spine tumor surgery can consist of delays in radiation therapy and delays in resumption or initiation of systemic treatment. Methods to decrease SSI in spine tumor surgery include plastic surgery closure, percutaneous instrumentation, pre-operative nutrition optimization, intrawound vancomycin powder (IVP), and betadine irrigation (BI) in combination or isolation [1-5].

Metastatic epidural spinal cord compression (MESCC) can be the first presentation of cancer and can also present in patients with an existing diagnosis of cancer whose disease progresses through treatment. In the presence of neurological deficits, most MESCC is managed surgically. Such urgent surgeries often do not allow for the assembly of a multidisciplinary team, including plastic surgery for wound closure. In addition, there is also not the opportunity to optimize nutritional status. Some intraoperative variables under the surgeon's control include the surgery's invasiveness, irrigation, and intrawound antibiotics.

The use of intrawound vancomycin powder (IVP) in isolation or combined with betadine irrigation (BI) has been described for spine tumor surgery, adult and pediatric deformity surgery, as well as in hip and knee arthroplasty [4-14]. A recent study of spine tumor patients evaluated SSI rates in patients receiving IVP and BI versus IVP only versus none (pre-incisional antibiotics only, non-BI irrigation)[4]. Interestingly, the IVP and BI groups had the lowest infection rates, and the IVP only and none groups had similar infection rates. This raises the possibility of a synergistic effect of IVP and BI in decreasing SSI. 

The objective of this study is to evaluate the risk factors for SSI in malignancy-related spinal infections and rates of infection observed in a single center with the use of BI and IVP. 

## Materials and methods

Study design

After obtaining IRB approval, spine tumor patients managed by one surgeon from 11/2012 to 11/2023 were identified using a surgical database (JotLogs, Efficient Surgical Apps, Portland, Maine). Patients were managed at tertiary hospitals carrying level I trauma designation and affiliated with cancer centers. Inclusion criteria were primary and metastatic spine tumor patients (extradural, mobile spine, and sacrum) receiving a combination of BI and IVP following and alive at 30 days post-op. Exclusion criteria were patients not receiving a combination of BI and IVP due to allergies, mortality within 30 days of surgery, and intradural tumors.

The following variables were collected: demographics (age, sex, race/ethnicity), tumor histology, history of pre-operative and post-operative radiation treatment, location of the tumor, type of surgery performed (instrumentation, type of decompression laminectomy only, transpedicular decompression, en-bloc resection, corpectomies), number of procedures per patient, SSI, wound culture results, and mortality at the latest follow-up.

Surgical site infection prophylaxis

All patients received intravenous (IV) antibiotics within an hour of incision, and intravenous antibiotics were continued for 24 hours. The most common presurgical IV antibiotics used were cephalosporins; however, clindamycin or vancomycin was used if the patient had a penicillin allergy. Betadine (0.3% weight volume) diluted with 0.9% normal saline was used as irrigation throughout the surgery (using the bur, irrigating after exposure, and irrigating before closure).

Intrawound vancomycin powder

Posterior cervical wounds received 500 mg of intrawound vancomycin powder, anterior cervical/thoracic/lumbar/sacral wounds received approximately 100 mg, and posterior thoracic/lumbar/sacral wounds received 1 g to 2 g.

Statistical analysis

The correlation between surgical site infection and potential risk variables was analyzed. Bivariant analysis used an independent t-test for continuous variables, and one-way analysis of variance (ANOVA) was utilized for multiple continuous variables. Fisher’s exact test to compare categorical variables. On univariant analysis, significant variables and those associated with an increased risk of infection were incorporated into a stepwise multivariable logistic regression model to identify independent risk factor variables related to infection. Statistical analysis was executed using Stata version 18 (StataCorp LLC, College Station, TX). Descriptive statistics of patient and surgical demographics are reported as mean, standard deviation, number, and percentage. The significance was set at p<0.05. Statistical power was analyzed using a post-hoc power analysis using G*Power 3.1. (Universität Mannheim, Mannheim, Germany) [15].

## Results

Demographics and tumor type

Over the study period, 102 patients undergoing 130 procedures meeting the inclusion criteria were identified out of a cohort of 108 patients. There were 19 primary spine tumors (18.6%) and 83 metastatic spine tumors (81.4%) (Table 1). At the latest follow-up, 52% of patients were alive. Demographics were an average age of 59.5 years old (range 7-92 years), 60.8% male, 39.2% female, 88.2% White, 9.8% Black, and 2% others (Table 1). Regarding the post-hoc statistical analysis, the cohort of study spine oncology patients had an effect size of d=0.5 and power=0.98. Therefore, it was considered that the result of this study has sufficient statistical power.

**Table 1:** Summarizes the patients' demographics and tumor type.

Demographics	Patients N (%)
Average age	59.5 years; range 7-92 years
Male	62 (60.8%)
Female	40 (39.2%)
White	90 (88.2%)
Black	10 (9.8%)
Others	2 (2%)
Metastatic	83 (81.4%)
Primary	19 (18.6%)

Tumor histology

The common primary spine tumors included six aneurysmal bone cysts (5.9%), three chordomas (2.9%), two aggressive hemangiomas (2%), one osteoblastoma (1%), one osteoid osteoma (1%), one hereditary multiple exostoses (1%), one angiolipoma (1%), one osteosarcoma (1%), one schwannoma (1%), one tumoral calcinosis (1%), and one radiation-induced sarcoma (1%) (Table 2). The most common metastatic histology included 18 lungs (17.6%), 11 prostate (10.8%), nine breast (8.8%), nine renal (8.8%), five colorectal (4.9%), four melanoma (3.9%), two endometrial (2%), and 10 others (9.8%) (Table 2). Hematologic malignancies included 10 multiple myelomas (9.8%) and three lymphomas (2.9%) (Table 2).

**Table 2:** Summarizes the tumor histology of the patient population.

Tumor histology	N (%)
Primary	
Aneurysmal bone cyst	6 (5.9%)
Chordoma	3 (2.9%)
Aggressive hemangioma	2 (2%)
Osteoblastoma	1 (1%)
Osteoid osteoma	1 (1%)
Hereditary multiple exostoses	1 (1%)
Angiolipoma	1 (1%)
Osteosarcoma	1 (1%)
Schwannoma	1 (1%)
Tumoral calcinosis	1 (1%)
Radiation-induced sarcoma	1 (1%)
Metastatic	
Lung	18 (17.6%)
Prostate	11 (10.8%)
Breast	9 (8.8%)
Renal	9 (8.8%)
Colon	5 (4.9%)
Melanoma	4 (3.9%)
Endometrial	2 (2%)
Other	10 (9.8)
Hematologic	
Multiple myeloma	10 (9.8%)
Lymphoma	3 (2.9%)

Surgical procedure 

**Figure 1:**
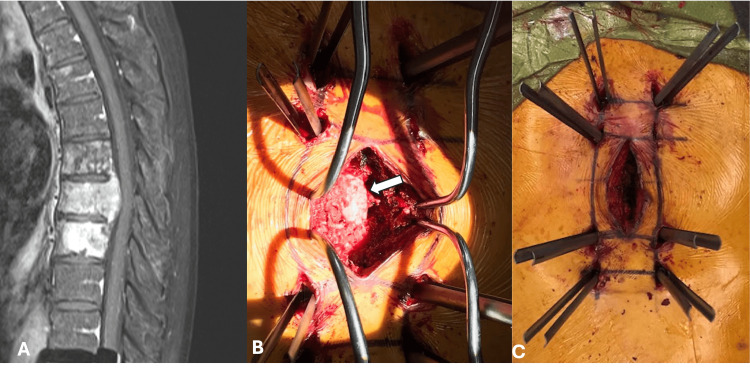
(A) Sagittal T1 MRI with contrast of a patient with metastatic prostate cancer with T8 epidural cord compression, (B) patient was managed with T8 bilateral transpedicular decompression with ligation of T8 nerve root (arrow), partial corpectomy, and (C) T6-T11 percutaneous instrumentation.

Most patients (85) underwent instrumentation (83.3%), with an average of 4.9 levels instrumented (range 3-16) (Table 3). Percutaneous instrumentation was used in 34 procedures (26.2%), and the rest had open procedures (Table 3). When percutaneous instrumentation was used, it was part of a hybrid technique where a midline incision was still made for decompression/transpedicular decompression (Figure 1). Instrumentation was not used in 21 procedures (16.2%). Transpedicular decompression was performed on 46 patients (45.4%), and anterior approaches were only used in 13 procedures (9.8%) (Table 3). Three total-en-bloc spondylectomies were performed, as well as three complete and partial sacrectomies for primary spine tumors.

**Table 3:** Summarizes the type of surgery performed (type of instrumentation, type of decompression, and the type of approach).

Type of procedure	N (%)
All instrumentation (open and percutaneous)	85 (83.3%)
Percutaneous only instrumentation	34 (26.2%)
Transpedicular decompression	46 (45.4%)
Anterior approach	13 (9.8%)

Surgical site infections

The post-op infection rate for our study group, patients receiving a combination of BI and IVP following spine tumor surgery, is 3.85% (5/130 procedures) (Table 4). All infections occurred in metastatic spine patients within four weeks of surgery, except one presented with an infection at 42 months post-op (Table 4). The one patient with delayed infection had prominent instrumentation that became exposed in the thoracic spine due to weight loss and kyphosis (Table 4). Cultures grew methicillin-resistant *Staphylococcus aureus *(MRSA) and Coagulase-negative Staphylococci in two patients (Table 4). Three patients had polymicrobial infections (two per patient), including (patient three) Strep, *Bacteroides fragilis*; (patient four) MRSA, Achromobacter; (patient five) methicillin-sensitive *Staphylococcus aureus* (MSSA), Corynebacterium. These infections were treated with irrigation, debridement, and IV antibiotics (Table 4).

**Table 4:** Age, tumor type, surgery performed, and culture results for the patients with surgical site infections. MIS: minimally invasive surgery, PSF: posterior spinal fusion, MRSA: methicillin-resistant *Staphylococcus aureus*, MSSA: methicillin-sensitive *Staphylococcus aureus*.

Patient no	Age and tumor type	Performance of surgery		Culture results
Patient 1	54 y/o M with metastatic lung cancer	Lesion at C7 initially treated with radiation therapy	Underwent C4-T3 PSF/laminectomy	Wound culture: Coagulase-negative Staphylococci
Patient 2	56 y/o M with adenoid cystic carcinoma	Initially managed with T8-T12 PSF, T10 corpectomy, and radiation therapy	Developed prominent implant with wound breakdown	Wound culture: MRSA
Patient 3	63 y/o F with endometrial cancer	Sacral lesion initially treated with radiation therapy	Underwent S1-S3 laminectomy and unilateral L4-pelvis instrumentation	Wound culture: Strep, Bacteroides fragilis
Patient 4	51 y/o M with adenocarcinoma of unknown origin	Sacral lesion initially treated with radiation therapy	Underwent L4-pelvis instrumentation for stability	Wound culture: MRSA, Achromobacter
Patient 5	68 y/o F with urothelial cancer	Prior radiation therapy to the thoracic spine	Underwent T3-T10 MIS instrumentation, T5-T8 laminectomy, T6 partial corpectomy, transpedicular decompression T6, T8	Wound culture: MSSA, Corynebacterium

Location of procedure

Most of the tumors and procedures were in the thoracic spine 41 (31.3%), followed by lumbar 29 (22.1%), thoracolumbar 18 (13.7%), cervical 18 (13.7%), sacral 14 (10.7%), and cervicothoracic 11 (8.4%) (Table 5).

**Table 5:** Shows the location where the procedures for the tumors were performed.

Tumor location	Patients N (%)
Thoracic	41 (31.3%)
Lumbar	29 (22.1%)
Thoracolumbar	18 (13.7%)
Cervical	18 (13.7%)
Sacral	14 (10.7%)
Cervicothoracic	11 (8.4%)

Plastic surgery soft tissue reconstruction

Initial wound closure with plastic surgery was performed in 14 patients (13.7%). Primary spine tumor patients were seven of these patients, and the other seven patients were metastatic spine patients. Despite only 20 patients with primary spine tumors, seven (35%) of these patients underwent initial plastic surgery closure due to the significant defects present. None of the primary tumor patients had an infection. One of the seven metastatic spine patients who had primary plastics closure had a delayed wound infection associated with prominent instrumentation. 

Pre-operative and post-op radiation status

Most patients, 50%, had post-op radiation treatment only, 15.7% had pre-operative radiation only, and 8.8% had pre- and post-op radiation therapy. 

Factors contributing to surgical site infections

All five patients with an SSI also had pre-operative radiation therapy. Pre-operative radiation therapy was significantly associated with the risk of SSI (p=0.005) (Table 6). Two of the five patients with an SSI had a procedure for sacral metastases; however, there was no significant difference in SSI rates (p=0.061) between patients with sacral tumors and those with tumors in non-sacral locations. The use of percutaneous instrumentation also did not lead to a significant difference in infection rates (p=0.139) (Table 6). There was no significant difference in infection rates between primary and metastatic tumors (p=0.58) (Table 6). A multivariable stepwise regression model incorporating the variables listed in Table 6 shows that pre-operative radiation is the sole independent risk variable for infection for patients diagnosed with a spinal tumor (OR: 18.1; 95%CI: 1.9-172.7; p=0.009). 

**Table 6:** Shows the risk variables associated with SSI for patients diagnosed with a spinal tumor. SSI: surgical site infection.

Variable	p-value
Pre-operative radiation	<0.005
Percutaneous vs. open instrumentation	0.139
Tumor type (primary or metastatic)	0.58

## Discussion

With the aging population in Western and East Asian countries and longer global life expectancies, the prevalence of cancer has increased. Due to innovations in systemic treatments and radiation therapy, cancer patients live longer. The spine is the most common site of metastasis within the skeletal system. SSI following spine tumor surgery leads to several setbacks to the patient, including hospital readmission, additional surgery, and delays in initiation of post-op radiation therapy, as well as systemic treatment. There are also expenses incurred by the healthcare system and potential impacts on hospital ratings due to infections and readmissions [16]. Metastatic spine surgery aims to preserve neurological function and maintain spinal stability. For primary malignant spine tumors, goals are often curative, and en-bloc resections may be needed to achieve this goal [17-19].

In this series of 102 spine tumor patients undergoing 130 procedures with one surgeon, concurrent administration of BI and IVP has led to a low rate of SSI (3.85%). Our results are comparable to what is found in the literature [4,9]. BI and IVP are inexpensive modalities that can be used in most operating rooms. Most of the patients had metastatic spine disease, and 18.6% had primary spine tumors. Nearly 26.2% of instrumentation was percutaneous, and 45.4% had transpedicular decompression to address ventral neural element compression or “separation surgery” [20].

In this series, pre-operative radiation therapy was a significant risk factor for SSI (p<0.005). In a series of 110 patients undergoing metastatic spine tumor surgery, Demura et al. reported an SSI rate of 7% [21]. The SSI rate for patients who had pre-operative radiation therapy was 32% as compared to 1.1% for those who did not have pre-operative radiation therapy. Recently, there have been conflicting reports on the role of pre-operative radiation therapy as a risk factor for SSI. In a multi-surgeon series of 205 patients, Vargas et al. reported no difference in wound complication rates between patients receiving pre-op radiation therapy (n=70), post-op radiation therapy (n=74), and those receiving no radiation at all (n=61) [22]. In a series of 297 patients undergoing metastatic spine tumor surgery, Sebaaly et al. reported an SSI rate of 5.1% with surgical time >4 or more hours and American Society of Anesthesiologists (ASA) >3 or higher were identified as independent risk factors for SSI [23].

Prior studies have reported conflicting results on the role of intrawound vancomycin powder in the prevention of SSI following spine tumor surgery. Okafor et al. reported a 4.9% infection rate with IVP in a mixed cohort of patients with primary and metastatic spine tumors [5]. Liu et al. compared 25 spine tumors not receiving IVP to 27 spine tumors receiving IVP and found no difference in infection rates [24]. In a series of 151 spine tumor patients, a comparison of SSI rates was performed between patients receiving None versus IVP only versus IVP/BI. The study found similar SSI in patients not receiving IVP (13%) as compared to patients receiving IVP (12.6%). However, the group receiving IVP/BI had a 2.7% infection rate [4]. This indicates that either BI by itself may be effective in reducing SSI or is synergistic with IVP.

There have been several studies reporting on the efficacy of IVP/BI in reducing SSI in spinal and orthopedic procedures. Mallet et al. evaluated the role of IVP/BI in SSI rates following surgery for adolescent idiopathic scoliosis (AIS) [9]. They had an overall SSI rate of 2.9% (n=9) from a series of 307 surgeries for AIS. The IVP/BI group has two infections (1.1%) vs. seven infections (5.4%) in the control group, p=0.04. They also noted lower rates of pseudarthrosis and instrumentation failure in the IVP/BI group (1.1%) compared to the control group (7%), p=0.01. The finding of lower pseudarthrosis in the IVP/BI group contrasts with the studies on the impact of betadine on osteoblasts, demonstrating the potential to inhibit bone growth [25].

Meza et al. compared SSI rates before and after the implementation of uniform use of betadine/IVP for AIS surgery in 740 patients [11]. They noted SSI rates decreased to 0.2% from 1.7% following the initiation of betadine/IVP (p<0.04). Roberto et al. also investigated the role of SSI before and after the implementation of IVP/BI protocols [14]. There were 1252 patients in the pre-IVP/BI implementation group and 1,173 in the IVP/BI implementation group. IVP/BI led to an SSI rate of 1.2% compared to 2.4% in the pre-implementation cohort (p=0.04). The study also noted that gram-positive organisms decreased from 80% in the pre-implementation group to 53% in the implementation group. 

Several methods to decrease SSI in spine tumor surgery have been reported, including pre-operative nutritional optimization, the use of plastic surgery for soft tissue reconstruction, the use of percutaneous implants, and IVP/BI. Especially in the setting of previously radiated wounds or revision spinal tumor surgery, collaboration with plastic surgery for soft tissue reconstruction is ideal [1]. Nguyen et al. reported that 102 patients (79 metastatic, 23 primary) underwent plastic surgery and soft tissue reconstruction [3]. Of the 102 patients, 37.3% had prior radiation therapy, and the majority (64.6%) had low albumin levels (<3.5). In this cohort, the use of plastic surgery for soft tissue reconstruction led to a 2.9% rate of wound complications needing re-operation. Percutaneous implants are also ideal for the metastatic spinal tumor population, where stabilization can be performed without a large soft tissue dissection, and tumor debulking/neural element decompression can be performed through a midline incision. Versteeg et al. reported on 101 patients, primarily neurologically intact, undergoing percutaneous stabilization [26]. They noted a 4% surgical site infection rate in their cohort. 

Lastly, the role of surgeon and hospital volume in managing spine tumors cannot be overstated. Williams et al. demonstrated using a New York State database that patients undergoing spine tumor surgery at low-volume hospitals had significantly higher odds of complications (p=0.03) [27]. Mortality within thirty days of hospital discharge was also significantly higher in low-volume hospitals (p=0.001). Schoenfeld et al., using a Florida database, noted surgery at low-volume hospitals and by low-volume surgeons was associated with surgical complications and higher readmission rates [28]. The lower SSI rate in this study may also be associated with a higher volume of spine tumor surgery by the first author as well as a higher volume of spinal tumors at the hospital.

Limitations

Our study had several limitations. This is a single surgeon series, and it is possible with additional surgeons participating, different conclusions may arise, and more patients may be enrolled. However, the benefit of a single surgeon series is the uniformity of the surgical approach and management principles. Second, the type of radiation therapy (stereotactic body radiation therapy, conventional radiation therapy) or treatment dosage was not collected. Third, some patients had completed metastatic spine-specific patient-reported outcomes (PROs); however, since all patients did not complete PROs, we did not include this analysis. Fourth, we did not uniformly use prognostic scores of survivals (Tomita, Tokuhashi, and Bauer) scores and did not evaluate these scores and their association with SSI [29]. Additionally, lab values, such as pre-operative albumin, were unavailable for all patients and not included in the analysis. Lastly, we did not have a comparator arm, so we adjusted for the confounders. However, this association of reduced infection rates when combining BI and IVP can be inferred.

## Conclusions

Surgical wound infections are a common complication following spine tumor surgery. This is a single surgeon series of 102 patients undergoing 130 procedures for primary and metastatic spine tumors. Pre-operative radiation therapy was a significant risk factor for SSI (p<0.005). Pre-operative radiation therapy was significantly associated with the risk of SSI (p=0.005). Percutaneous instrumentation did not lead to a significant difference in infection rates (p=0.139). There was no significant difference in infection rates between primary and metastatic tumors (p=0.58). The synergist use of BI and IVP led to an infection rate of 3.85%, which is one of the lowest in the literature.

Several studies have reported the benefits of IVP and BI in various spine and orthopedic procedures. These are inexpensive modalities that can be implemented in most settings. Future randomized prospective studies are needed to evaluate further the role of BI in isolation and in combination with IVP in decreasing SSI rates following spine tumor surgery. 
